# Regression of sporadic intra-abdominal desmoid tumour following administration of non-steroidal anti-inflammatory drug

**DOI:** 10.1186/1477-7819-6-17

**Published:** 2008-02-08

**Authors:** Keita Tanaka, Reigetsu Yoshikawa, Hidenori Yanagi, Makoto Gega, Yoshinori Fujiwara, Tomoko Hashimoto-Tamaoki, Syozo Hirota, Tohru Tsujimura, Naohiro Tomita

**Affiliations:** 1 Department of Surgery, Hyogo College of Medicine, Nishinomiya, Hyogo, Japan; 2 Institute for Advanced Medical Sciences, Hyogo College of Medicine, Nishinomiya, Hyogo, Japan; 3 Department of Genetics, Hyogo College of Medicine, Nishinomiya, Hyogo, Japan; 4 Department of Radiology, Hyogo College of Medicine, Nishinomiya, Hyogo, Japan; 5 Department of Pathology, Hyogo College of Medicine, Nishinomiya, Hyogo, Japan

## Abstract

**Background:**

Desmoid tumours or fibromatoses are rare entities characterized by the benign proliferation of fibroblasts, which can be life-threatening due to their locally aggressive properties. Surgery is widely accepted as the first line of treatment for extra-abdominal desmoids; however, it is not recommended for intra-abdominal desmoids because of the high-risk of recurrence and difficulties with the operation. Here, we report on a patient with sporadic intra-abdominal desmoid tumours, who showed partial response following the intake of non-steroidal anti-inflammatory drugs.

**Case presentation:**

A 73-year-old man presented with swelling and pain of the right leg. Computed tomography showed an abnormal multilocular soft-tissue mass (95 × 70 mm) in the right pelvis, which was revealed by biopsy to be a desmoid tumour. Immunohistochemical analysis showed that the tumour cells expressed vimentin, but not smooth-muscle actin, CD34, or desmin. Very few Ki-67-positive cells were found. Non-cytotoxic treatment with etodolac (200 mg/day) was chosen because of the patient's age, lack of bowel obstruction, and the likelihood of prostate cancer. Two years after the commencement of non-steroidal anti-inflammatory drug administration, computed tomography showed a decrease in tumour size (63 × 49 mm), and the disappearance of intratumoural septa.

**Conclusion:**

Our case report suggests that non-steroidal anti-inflammatory drug treatment should be taken into consideration for use as first-line treatment in patients with sporadic intra-abdominal desmoid tumours.

## Background

Desmoid tumours or aggressive fibromatoses are rare soft tissue neoplasms that can occur sporadically or in association with familial adenomatous polyposis (FAP). These tumours are aggressive, infiltrative, and destructive, and can recur frequently, although they do not metastasise [1]. The aetiology of these tumours is unknown, but genetic, hormonal (*e.g*., deterioration triggered by pregnancy), and physical factors (*e.g*., previous surgery) play a role in their development and growth. A distinction is often made between desmoids in patients with FAP and those in patients without FAP, but clinically these tumours are treated the same; the only difference is the preferential intra-abdominal location of FAP desmoids.

Surgery is the mainstay treatment for extra-abdominal and abdominal-wall desmoids; however, is not recommended for intra-abdominal desmoids because of the high-risk of recurrence and the difficulties associated with the operation. Recently, we have shown that the chemotherapeutic modality of doxorubicin plus dacarbazine is efficacious and safe for desmoid patients with FAP [2]. After all, the main aim of desmoid treatment is local control. Several pharmacological agents have successfully been used to treat desmoids, including anti-oestrogen and non-steroidal anti-inflammatory drugs (NSAIDs) [1]. NSAIDs efficiently block cyclooxygenase (COX) activity and are well known to be beneficial in the prevention of colorectal carcinogenesis including FAP.Here, we report on a patient with sporadic intra-abdominal desmoid tumours, who underwent non-cytotoxic NSAID therapy and showed remarkable regression. To our knowledge, this is the first report demonstrating the potency of NSAIDs for both FAP-associated desmoids and sporadic desmoid tumours.

## Case presentation

**Figure 1:**
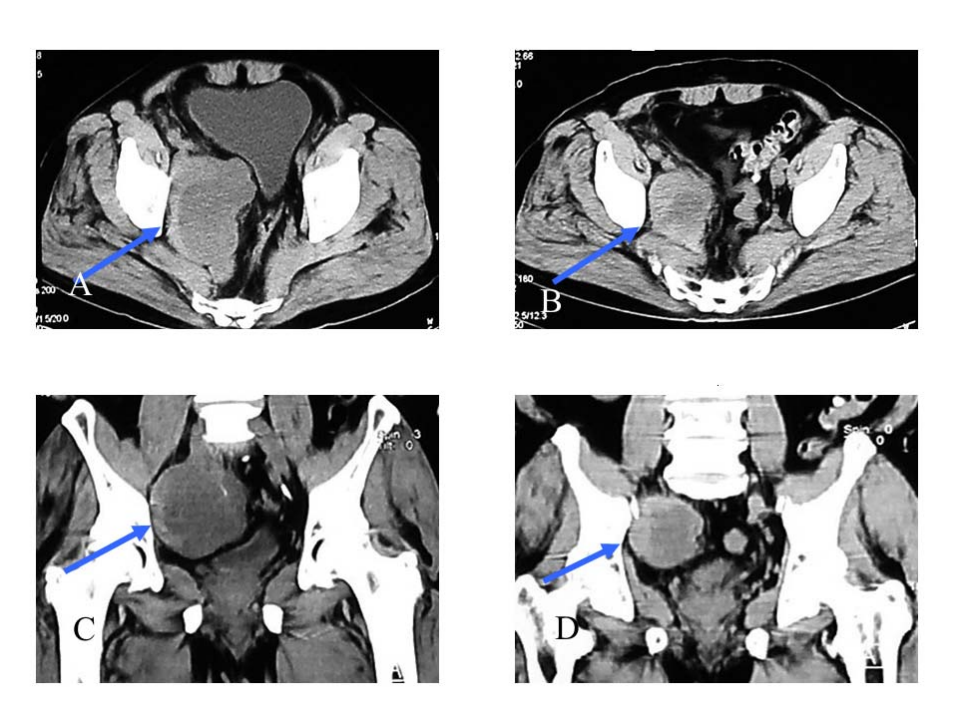
**Desmoid tumour before (A, C) and 2 years after (B, D) the commencement of NSAID**. Multi planner reformation (MPR)-CT demonstrates the sporadic desmoid tumours originating from the intra-abdominal cavity (arrows). Frontal (A, B) and axial (C, D) images are shown. The tumour has shown a remarkable shrinkage with a regression rate of 68.5% along with disappearance of intratumoural septa.

A 73-year-old man presented with pain and swelling of the right leg. Computed tomography (CT) and magnetic resonance imaging (MRI) showed an abnormal multilocular soft tissue mass (95 × 70 mm) in the right pelvis, which was suspected of lymphoma or lymph node metastasis (Figure 1). The patient had not undergone previous surgery, had no family history of colorectal cancer or polyps, and showed no abnormality on colonoscopy. On clinical admission, a CT-guided biopsy revealed the intra-abdominal mass to be a desmoid tumour. Non-cytotoxic treatment was chosen because of the patient's age, lack of bowel obstruction, and the likelihood for prostate cancer. Initial treatment commenced with administration of the COX-2 inhibitor, meloxicam. However, the patient experienced hot flushes, so treatment was changed to an alternative COX-2 inhibitor, etodolac (200 mg/day). After two years of the commencement of etodolac, CT showed a decrease in tumour size (to 63 × 49 mm) along with disappearance of intratumoural septa. Regression rate of partial response (PR) was 68.5%, and no adverse events were reported.

### Histological examination

**Figure 2:**
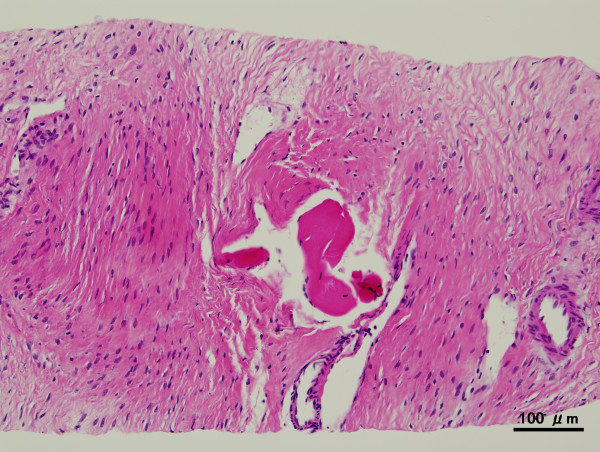
**Microscopic examination of biopsy specimen**. Spindle-cellular tumours surrounded muscle components. The tumour cells had pale eosinophilic cytoplasms and chromatin structures, and were embedded in a collagen network interrupted by fibrotic sections. Scale bar represents 100 μm.

Microscopic examination of the biopsy specimen revealed spindle-cellular tumours surrounding muscular elements. The tumour cells had a pale eosinophilic cytoplasm and chromatin structures, and were embedded in a collagen network interrupted by fibrotic sections (Figure 2). Immunohistochemical analysis showed that the tumour cells expressed vimentin, but not smooth-muscle actin (SMA), CD34, or desmin. Very few Ki-67-positive cells were found. After the diagnosis of desmoid tumour, analysis of β-catenin expression could not be undertaken because of an insufficient sample volume.

## Discussion

In patients with FAP, desmoid tumours are caused by a mutation of the *adenomatous polyposis coli *(*APC) *gene [3]. By contrast, 75% of desmoid tumour patients without FAP harbour a somatic mutation in either the *APC *or β*-catenin *genes, resulting in β-catenin protein stabilisation [3-5]. Several NSAIDs have been shown to inhibit the activity of β-catenin-dependent reporter genes in malignant cell lines, and to induce β-catenin degradation [6,7]. Moreover, NSAIDs appear to inhibit the initial stages of the colorectal adenoma-carcinoma sequence, suggesting a link to the APC/β-catenin/TCF pathway (Wnt-signalling pathway), and the colonic polyps of patients treated with NSAIDs demonstrate reduced nuclear accumulation of β-catenin [6]. Oncogenic activation of the Wnt-signalling pathway by mutations in *APC *or β*-catenin*, which results in the accumulation and nuclear translocation of β-catenin and in β-catenin/TCF4-regulated transcription of TCF target genes, is mandatory for the initial neoplastic transformation of intestinal epithelium [8,9]. The basic and clinical data imply that NSAIDs inhibit β-catenin activity or its stability. Theoretically, NSAIDs may, hence, have the potency to inhibit the development of some desmoid tumours *via *interference of β-catenin function, although the biochemical basis for these effects has not been clarified.

The treatment of desmoid tumours remains enigmatic despite longstanding investigation. However, it appears that analysis of APC/β-catenin expression in desmoid tumours might determine the efficacy of NSAIDs, and contribute to tumour-growth inhibition and survival in desmoid patients with β-catenin protein stabilisation. Surgical treatment is difficult and requires a wide resection margin to prevent tumour recurrences. Desmoid tumours are locally invasive lesions that do not metastasizes; thus, a decrease in the growth rate could prevent the need for more radical treatments, which would be beneficial for elderly patients in poor general condition. At our institution, a chemotherapeutic regimen of doxorubicin plus dacarbazine is the preferred first-line treatment for FAP-associated unresectable intra-abdominal desmoids. However, its application is restricted to patients with symptoms of bowel obstruction. Other patients are treated initially with COX-2 inhibitors. In the present case, the tumour demonstrated PR following treatment with etodolac, even though the COX-2 selectivity of this NSAID is far weaker than that of meloxicam. The patient was offered no additional therapy, and remained asymptomatic even after two year of follow-up, without any evidence of deterioration. This suggests that the COX-2 selectivity of NSAIDs might not be critical for determining inhibitory effect against desmoid tumours.

Surgery is the treatment of choice for patients with desmoids loco-regionally confined to the body wall. However, surgical excision provides for only a narrow therapeutic window, when desmoid tumours are located in the abdominal cavity and recur even if they are not associated with FAP. Therefore, the efficient blockade of β-catenin by NSAIDs might be useful in achieving significant and durable cytoreduction, obviating the need for surgical intervention in patients with sporadic desmoid tumour as well as those with FAP-associated desmoids, especially when they show a high risk of operation (general condition, age, complication, quality of life, *etc*.) Continued efforts at improving the efficacy of such regimens with possible addition of novel molecule-targeting agents should be made in the future.

## Conclusion

This is the first report of a patient with sporadic desmoid tumour who has shown PR following the administration of an NSAID alone. Our case report suggests that NSAID treatment should be considered for use as a first-line treatment in patients with sporadic intra-abdominal desmoid tumours and a high risk general condition, as well as those with FAP-associated desmoids.

## Competing interests

The author(s) declare that they have no competing interests.

## Authors' contributions

**KT **participated in the preparation of the manuscript, and carried out the immunohistochemical analysis. **RY **conceived and designed the study, and drafted the manuscript. **HY **conceived the study, and edited the manuscript for its scientific content. **MG **participated in the preparation of the manuscript. **YF **participated in the evaluation of the immunohistochemical study. **TH-T **participated in the study design and coordination. **SH **accomplished CT-guided biopsy. **TT **was responsible for the evaluation of the immunohistochemical study, and participated in the study design and coordination. **NT **edited the manuscript for its scientific content. All authors read and approved the final study.
